# Cost-effectiveness of digoxin versus beta blockers in permanent atrial fibrillation: the Rate Control Therapy Evaluation in Permanent Atrial Fibrillation (RATE-AF) randomised trial

**DOI:** 10.1136/heartjnl-2024-324761

**Published:** 2025-01-16

**Authors:** Zainab Abdali, Karina V Bunting, Samir Mehta, John Camm, Kazem Rahimi, Mary Stanbury, Sandra Haynes, Dipak Kotecha, Sue Jowett

**Affiliations:** 1 Health Economics Unit, School of Health Sciences, University of Birmingham, Birmingham, UK; 2 Department of Cardiovascular Sciences, University of Birmingham, Birmingham, UK; 3 NIHR Birmingham Biomedical Research Centre, Birmingham, UK; 4 Birmingham Clinical Trials Unit, School of Health Sciences, University of Birmingham, Birmingham, UK; 5 Cardiology Clinical Academic Group, St George's University of London, London, UK; 6 Deep Medicine, Nuffield Department of Reproductive and Women’s Health, University of Oxford, Oxford, UK; 7 Patient and Public Involvement Team, RATE-AF Trial, University of Birmingham, Birmingham, UK; 8 NHS West Midlands Secure Data Environment, University Hospitals Birmingham NHS Foundation Trust, Birmingham, UK

**Keywords:** Atrial Fibrillation, Health Care Economics and Organizations, Quality of Health Care

## Abstract

**Background:**

Atrial fibrillation (AF) is a major and increasing burden on health services. This study aimed to evaluate the cost-effectiveness of digoxin versus beta-blockers for heart rate control in patients with permanent AF and symptoms of heart failure.

**Methods:**

RAte control Therapy Evaluation in permanent Atrial Fibrillation (RATE-AF) was a randomised, open-label, blinded, endpoint trial embedded in the UK National Health Service (NHS) to directly compare low-dose digoxin with beta-blockers (ClinicalTrials.gov: NCT02391337). A trial-based cost-utility analysis was performed from a healthcare perspective over 12 months. Resource use in primary and secondary healthcare services, medications and patient-reported quality of life were prospectively collected to estimate differences in costs and quality-adjusted life years (QALYs).

**Results:**

RATE-AF randomised 160 patients with mean age of 76 (SD 8) years and 46% women, of which 149 patients (n=73 digoxin, n=76 beta blockers) had complete data and survived to 12-month follow-up. Treatment with digoxin was significantly less costly, with a mean saving of £530.41 per patient per year (95% CI −£848.06 to −£249.38, p=0.001). This was principally due to substantially lower rates of adverse events, with less primary and secondary healthcare utilisation compared with beta-blocker therapy. There was no significant difference in QALYs (0.013; 95% CI −0.033 to 0.052, p=0.56). At the £20 000 per-QALY willingness to pay threshold, the probability of digoxin being cost-effective compared with beta-blockers was 94%, with potential annual savings to the NHS of £102 million/year (95% CI £48 million to £164 million saving, p=0.001).

**Conclusions:**

Digoxin is a less costly option when compared with beta-blockers for control of heart rate in suitable patients with permanent AF, with larger cost-effectiveness studies warranted to advise on national and global policy-making.

**Trial registration number:**

NCT02391337, EudraCT 2015-005043-13.

**WHAT IS ALREADY KNOWN ON THIS TOPIC**

- Atrial fibrillation (AF) is doubling in prevalence every few decades and is associated with considerable cost for healthcare services.
- The RAte control Therapy Evaluation in permanent Atrial Fibrillation (RATE-AF) trial showed that heart rate control with digoxin and beta blockers in patients with permanent AF and heart failure can improve symptoms and functional capacity.

**WHAT THIS STUDY ADDS**

- The economic analysis from a healthcare perspective within a randomised trial showed that digoxin is associated with less cost than conventional first-line beta-blocker therapy, mainly from fewer serious adverse events, with uncertain effect on quality-adjusted life years (QALY).
- Digoxin dominated beta-blockers, with 94% probability of digoxin being more cost-effective at a willingness-to-pay threshold of £20 000 per QALY gained.

**HOW THIS STUDY MIGHT AFFECT RESEARCH, PRACTICE OR POLICY**

- Extrapolation to current costs for AF within the UK National Health Service suggests considerable potential savings using digoxin as initial therapy in this patient group.
- While likely applicable to similar healthcare settings outside the UK, further studies with formal economic evaluation are needed to address this key evidence gap and the implications for global management of patients with AF.

## Introduction

 Atrial fibrillation (AF) is the most common cardiac arrhythmia and a major risk factor for thromboembolic disease such as stroke and vascular dementia.^1^ Prevalence of AF is predicted to double over the coming decades^2^ due to an ageing population and better methods of detection. AF currently accounts for over 1% of the annual UK National Health Service (NHS) budget, predominantly from hospital admissions.^3^ Although half of patients with AF have ‘permanent’ AF (where clinicians do not pursue conversion to normal sinus rhythm), there are very few interventions in this population evaluated with randomised controlled trials (RCTs).^4^ Medications to control the rapid heart rate in AF have been shown in historical studies to be cost-effective.^5^ However their contemporary value is unknown, particularly as many of those with AF in current practice also suffer from heart failure that can impact on treatment choice and effectiveness.^6^

The RAte control Therapy Evaluation in permanent Atrial Fibrillation (RATE-AF) trial was codesigned and comanaged by a Patient and Public Involvement (PPI) team,^7^ and the first head-to-head RCT of rate control agents in patients with permanent AF and symptoms of heart failure. RATE-AF compared low-dose digoxin (typically used as a second-line medication) with beta- blockers (usual first-line approach). No difference was found in the primary outcome of health-related quality of life (QoL) in the physical activity domain at 6 months, although nearly all secondary outcomes favoured digoxin by 12 months, with better patient functional capacity and less evidence of cardiac strain. There were lower rates of side effects, cardiovascular events and hospital admissions in those randomised to digoxin.^8^ This prespecified health economic analysis of the RATE-AF trial evaluates the cost-utility of digoxin compared with beta-blockers for heart rate control from a healthcare perspective.

## Methods

### The RATE-AF trial

The clinical trial design, protocol and outcomes have previously been published.^4 8^ In brief, RATE-AF was a pragmatic, NHS-embedded, open-label, blinded, endpoint RCT in patients with permanent AF and symptoms of heart failure in need of heart rate control. All participants provided written informed consent after review of participant information material created by the PPI team. The trial was publicly funded by the UK National Institute for Health and Care Research (CDF-2015-08-074); registration ClinicalTrials.gov (NCT02391337) and clinicaltrialsregister.eu (EudraCT 2015-005043-13).

### Participants and randomisation

Recruitment occurred from primary care practices and three hospitals in the West Midlands region of England (2016–2018). Patients had to be diagnosed with permanent AF, aged 60 years or older and with symptoms of possible heart failure with breathlessness equivalent to New York Heart Association class II or above. Exclusion criteria were limited to ensure the population enrolled reflected routine clinical practice (see online supplemental table 1 for full eligibility criteria). Participants were randomised using a telephone or web-based portal to either digoxin (62.5–250 µg daily) or a beta-blocker (bisoprolol 1.25–10 mg daily) in a 1:1 ratio. Alternative beta- blockers were acceptable for those with intolerance to bisoprolol. Note that both randomised groups received an intervention, as necessitated by their clinical status. The vast majority of patients in usual standard care in the UK would receive beta-blockers, with digoxin typically reserved as a second-line agent for patients intolerant or not responding to beta-blocker therapy. Randomisation used a minimisation algorithm to ensure balance between the treatment groups for gender and modified European Heart Rhythm Association (mEHRA) functional class score.

### Economic analysis overview

The within-trial economic analysis estimates the cost-utility of digoxin versus beta-blockers for heart rate control from a UK NHS perspective. Costs are expressed in British pound sterling (2019/2020 price year) and health outcomes in quality-adjusted life years (QALYs), as recommended by the National Institute for Health and Care Excellence (NICE).^9^ The time horizon covered the period from randomisation to the end of follow-up at 12 months, and all data included in the analysis were collected via study data collection forms and self-reported questionnaires. Differences in mean costs and QALYs between treatment arms were used to calculate the incremental cost-effectiveness ratio, or to determine dominance if a comparator was found to be more effective and less costly,^10^ with NICE thresholds of £20 000 and £30 000 per QALY gained.^9^

Costs and outcomes were not discounted as the trial was limited to 12 months of follow-up. NICE recommendations were used to guide methods^9^ and findings reported in accordance with the Consolidated Health Economic Evaluation Reporting Standards. The health economists performing this analysis were independent of the RATE-AF team that deployed the trial.

### Resource use and cost

Resource use data were collected prospectively for individual patients, covering primary healthcare, secondary healthcare and medication use. Primary and secondary healthcare resources were considered in the analysis if they were related to AF or other cardiovascular events, in consultation with clinical experts. Data on primary and secondary healthcare service utilisation were collected at the 6 and 12-month follow-up visits using specific case report forms by a study research nurse not involved in the usual clinical care of each patient. All adverse events (as listed in the Summary of Product Characteristics for each drug) were compiled by directly asking participants at each trial visit and review of the electronic medical record. The number and the type of primary care contacts, including general practitioner (GP), nurse or allied health professionals, were involved in discussion with the patient and updated with information from the primary care team. Cardiovascular events and any serious adverse events (SAE) underwent a process of independent clinical adjudication.

SAEs were costed according to the Healthcare Resource Group (HRG) code that included the costs of medical management incurred. An average of the HRG unit costs for each clinical inpatient event was calculated from NHS reference costs.^11^ To reflect complicated cases with prolonged hospitalisation, the high-end HRG cost that corresponded to the cardiovascular event was used. Hospital stay was assumed to be prolonged if the patients spent more than 10 days in the hospital for a specific cardiovascular event. It was assumed that patients who died of cardiovascular events did not consume any resources after the date of death, therefore missing cost values were replaced with zero. Costs of trial and additional medications were estimated using the average cost of the daily dose multiplied by the average duration of receiving the treatment. Unit costs for all types of health service resources were obtained from Personal Social Services Research Unit, NHS reference costs and the British National Formulary (online supplemental table 2). Unit costs were assigned to health service resources to generate total healthcare costs per patient over 12 months of follow-up.^10^

### Health outcomes

Patient-reported QoL using the EuroQol 5-Domain 5-Level (EQ-5D-5L) questionnaire was the primary health outcome measure for the economic analysis. Patients completed the questionnaire at baseline and 6 and 12 months of follow-up. Individual patient responses were used to calculate health index scores applying the UK crosswalk value set. Score values range from 1 (state of perfect health) to 0 (state of death), with negative scores representing a health state that is worse than death. The area under the curve approach was employed to calculate QALYs based on patient-specific utility scores at each time point over the 12-month period.^10^ For patients who died due to a cardiovascular event, a utility value of zero was assigned based on the date of death.

### Statistical and economic evaluation analyses

The base case analysis was conducted using complete data on both resource use and health outcomes at baseline, 6 months and 12 months. For health outcomes, patient responses to all EQ-5D-5L items at all follow-up points were required for the analysis to generate QALYs, otherwise the data were defined as missing. Similarly, data on resource use were deemed to be missing if any of the follow-up forms were not completed. QALYs, resource use and cost per patient were estimated and presented as means and SD by treatment arm. As cost and health outcome data are likely to be skewed, mean differences between treatment arms with the 95% CIs were calculated from 1000 resamples using bias-corrected and accelerated bootstrapping. The difference in QALYs was adjusted for baseline EQ-5D-5L score, mEHRA and left ventricular ejection fraction (LVEF; analysed from blinded cardiac ultrasound), in addition to age and gender.^12^ The mean difference in total costs was adjusted for baseline mEHRA and LVEF scores, age and gender.

To account for uncertainty due to sampling, a bootstrapping approach was used to generate 5000 paired estimates of differences in mean costs and health outcomes (QALYs). Estimates were presented as a scatterplot on a cost-effectiveness plane.^13^ The net monetary benefits were generated from these estimates to calculate the probability of digoxin being cost-effective at different threshold values of willingness to pay per additional QALY, with results plotted as a cost-effectiveness acceptability curve.^14^ A multiple imputation approach was employed to account for the impact of missing data on the results.^15^ Missing values were predicted from the observed values using chained equations with predictive mean matching.^15 16^ The imputation model included gender, age, baseline mEHRA class and LVEF for cost data, and the same variables plus baseline EQ-5D-5L score for QALYs. Treatment allocation was also included as a variable in all multiple imputation models. 10 imputed datasets were used, reflecting the percentage of incomplete cases^17^ and Rubin’s rule used to combine the imputed datasets into one final imputed variable.^18^ An additional analysis was conducted according to the intention-to-treat principle including all randomised patients. The imputed data of costs and QALYs were used to determine cost-effectiveness.

The health economic analysis was prespecified in the RATE-AF trial protocol prior to the inclusion of any participants. Extrapolation from the RATE-AF trial to an annual UK NHS cost for AF was calculated post hoc using published sources of data on AF prevalence, medication usage and the cost of managing AF to the NHS (online supplemental table 4).^317 19^,^21^ A two-tailed p value of <0.05 was considered statistically significant, with data analysis carried out using Stata (V.17.0; StataCorp, Texas).

## Results

160 patients were randomised, with mean age at enrolment of 76 (SD 8) years and 46% women (table 1). A total of 11 patients died; four in the digoxin group and seven with beta-blockers. There were no toxicity events recorded with the low-dose digoxin approach. Data on EQ-5D-5L and resource use at all time points (baseline, 6 months and 12 months) were available for 149 patients (93%), with 73 randomised to digoxin and 76 to beta blockers.

**Table 1:** Baseline characteristics

Characteristic	Digoxin(n=80)	Beta-blockers(n=80)
Age, mean years (SD)	74.5 (8.3)	76.8 (8.1)
Gender, women, n (%)	36 (45.0)	38 (47.5)
White British/Irish ethnicity, n (%)	75 (93.8)	74 (92.5)
Treatment for hypertension, n (%)	56 (70.0)	60 (75.0)
Diabetes mellitus, n (%)	16 (20.0)	22 (27.5)
Heart rate, mean beats per minute (SD)	100.1 (16.8)	99.2 (19.2)
New York Heart Association functional class, mean (SD)	2.4 (0.5)	2.4 (0.6)
Modified European Heart Rhythm Association class 3 (severe) or class 4 (disabling) symptoms, n (%)	43 (53.8)	37 (46.3)
Left ventricular ejection fraction, mean % (SD)	56.2 (8.8)	57.6 (10.5)
Systolic blood pressure, mean mm Hg (SD)	134.2 (14.7)	137.1 (17.5)
SF-36 physical component summary score*, mean (SD)	28.9 (11.6)	27.2 (10.2)
EQ-5D-5L summary index score, mean (SD)	0.67 (0.19)	0.63 (0.22)
EQ-5D-5L visual analogue scale, mean (SD)	64.0 (16.6)	61.6 (20.3)

* Normalizsed for the UK population, with a score of 50 being the expected normal score.

EQ-5D-5L, EuroQol 5-Domain 5-Level; SF-36, 36-Item Short Form Survey.

### Resource use and costs

Details of the average healthcare resource use and costs over 12 months are presented in table 2. The mean total costs over the trial period were £46.19 per patient randomised to digoxin and £535.39 per patient for those randomised to beta blockers. The adjusted bootstrapped difference in favour of digoxin was −£530.41 (95% CI −£848.06 to −£249.38, p=0.001).

**Table 2:** Resource use and associated per-patient costs (complete case data)

Resource use	Digoxin (n=74)		Beta-blockers (n=76)		Mean bootstrapped per-patient cost difference (£; 95% CI) and P value for digoxin versus beta blockers*
	Mean unit (SD)	Mean per-patient cost (£) (SD)	Mean unit (SD)	Mean per-patient cost (£) (SD)	
**Primary care**					
General practitioner contact					
Face to face	0.21 (0.62)	8.06 (24.43)	0.72 (0.92)	28.39 (36.01)	−19.13 (−28.08 to −10.37)
Home	–	–	0.01 (0.11)	1.32 (11.54)	−0.73 (−3.15 to 0)
Nurse contact					
Face to face	0.03 (0.16)	0.30 (1.78)	0.07 (0.30)	0.71 (3.24)	−0.40 (−1.23 to 0.28)
Home	–	–	0.01 (0.11)	0.23 (2.01)	−0.16 (−0.61 to 0.00)
Total primary care costs		8.36 (24.87)		30.66 (40.44)	−22.43 (−31.05 to −10.52); p<0.001
**Secondary care**					
Heart Failure	–		0.05 (0.22)	133.58 (641.88)	−164.28 (−397.24 to −11.58)
Arrhythmia	0.01 (0.12)	8.88 (75.84)	0.01 (0.11)	44.82 (390.69)	−24.24 (−111.25 to 17.88)
Chest pain	–	–	0.04 (0.20)	15.99 (79.39)	−19.09 (−46.16 to 0.00)
Stroke	–	–	0.03 (0.16)	124.13 (760.08)	−140.53 (−396.03 to 0.00)
Endocarditis	–	–	0.01 (0.11)	21.22 (185.02)	−15.80 (−63.26 to 0.00)
Myocardial Infarction	–	–	0.01 (0.11)	16.43 (143.27)	−18.58 (−66.83 to 0.00)
Syncope	–	–	0.01 (0.11)	43.24 (376.93)	−42.67 (−158.14 to 0.00)
Pacemaker implantation	–	–	0.04 (0.20)	85.42 (435.09)	−92.85 (−213.48 to 0.00)
Total secondary care costs		8.88 (75.84)		484.83 (1266.02)	−518.04 (−865.19 to −247.63); p=0.001
**Medication**					
Trial medication		23.49 (11.35)		18.34 (16.40)	4.56 (−1.11 to 8.87)
Additional/concomitant medication		5.47 (21.08)		1.57 (13.67)	3.50 (−0.99 to 8.48)
Total health service cost		46.19 (87.19)		535.39 (1261.21)	−530.41 (−848.06 to −249.38); p=0.001

Costs are presented in British pound sterling (GBP) 2019/2020 prices for the 12 months following randomisation. Only includes patients with complete resource use and outcome data used in the main health economic analysis. Bootstrapped costs are adjusted for baseline class, left ventricular ejection fraction, age and gender; favour digoxin in comparison to beta-blockers.

n denotes number of participants.

* Bootstrapped costs are adjusted for baseline modified European Heart Rhythm Association class, left ventricular ejection fraction, age and gender; values <0 favour digoxin in comparison to beta-blockers.

**Figure 1:**
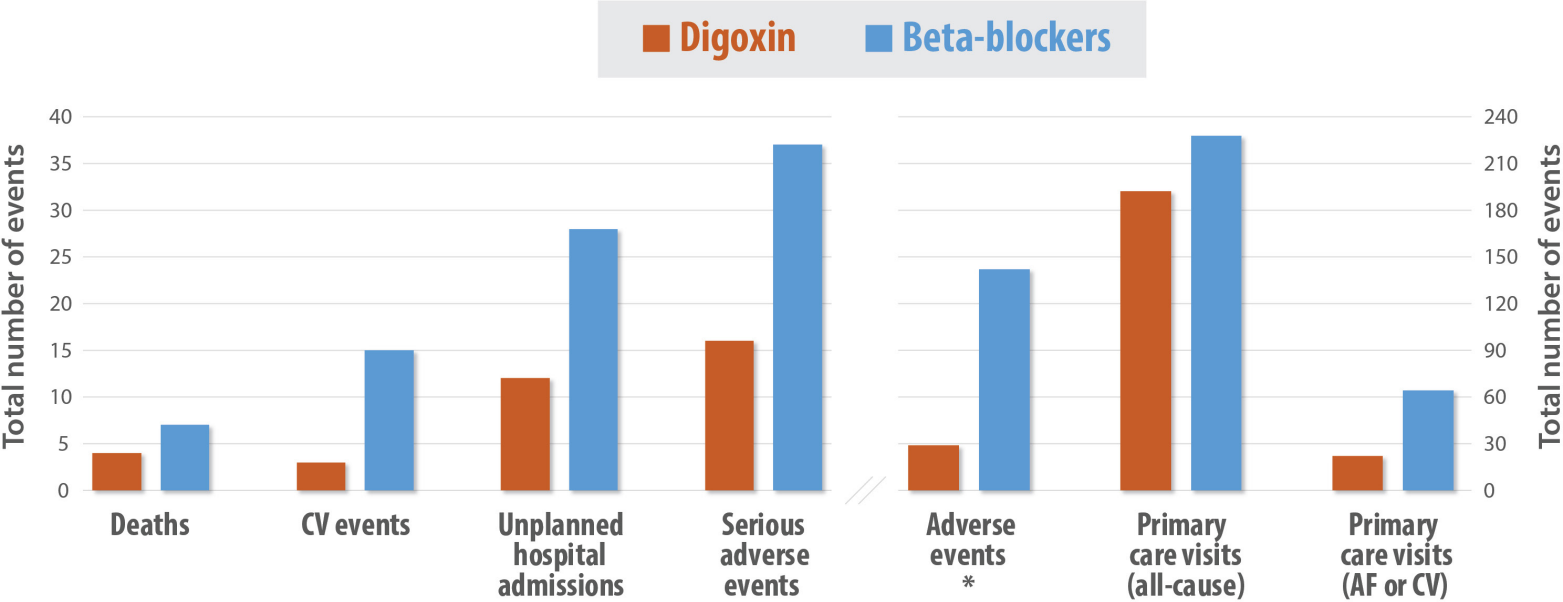
RAte control Therapy Evaluation in permanent Atrial Fibrillation (RATE-AF) trial adverse outcomes for digoxin versus beta-blockers. Events recorded for the 12-month follow-up period in patients randomised to digoxin or beta-blockers. Serious adverse events and cardiovascular events were independently adjudicated. *A prespecified statistical analysis was performed for adverse treatment events (p<0.001). AF, atrial fibrillation; CV, cardiovascular.

Patients randomised to digoxin had less frequent face-to-face GP and nurse visits, and the mean total primary care cost was −£22.43 per patient over the 12-month period compared with those randomised to beta-blockers (95% CI −£30.12 to −£10.20, p<0.001). The cost of secondary care services, mainly inpatient care, was significantly lower in the digoxin arm, reﬂecting that these patients had substantially less SAEs (16 in 13 patients for digoxin vs 37 in 21 patients for beta- blockers) and less treatment-related adverse events (29 in 20 patients for digoxin vs 142 in 51 patients for beta-blockers) (figure 1 and online supplemental table 3). The mean total costs for secondary care were £8.88 (SD £75.84) per patient over 12 months in the digoxin group and £484.83 (SD £1266.02) per patient in the beta-blocker group, with adjusted bootstrapped difference of −£518.04 per patient in favour of digoxin (95% CI −£865.19 to −£247.63, p=0.001). There were a number of high-cost outliers, especially for the secondary care services, leading to a skewed distribution of costs. The cost of trial medications, additional therapy and concomitant drugs were not significantly different between treatment groups.

### Health outcomes

EQ-5D-5L scores and QALYs are presented in table 3. An increase in mean EQ-5D-5L score from baseline to 12 months was seen for participants in both randomised arms, with no significant difference between digoxin and beta -ockers for the index score, and a significantly better visual analogue scale (VAS) score in those randomised to digoxin (mean bootstrapped difference 7.96, 95% CI 1.45 to 14.05, p=0.013). There were no significant differences between treatments in QALYs (0.013, 95% CI −0.033 to 0.052, p=0.56).

**Table 3:** Health utility outcomes by treatment arm

Health utility	Digoxin	Beta-blockers	Mean bootstrapped difference (95% CI) and P value for digoxin versus beta-blockers*
**EQ-5D-5Lbaseline**			
n	80	80	
Mean index score (SD)	0.665 (0.195)	0.627 (0.218)	0.029 (−0.026 to 0.086); p=0.32
Mean VAS score (SD)	63.88 (16.73)	61.63 (20.34)	1.88 (−2.77 to 7.79); p=0.50
**EQ-5D-5L6months**			
n	76	76	
Mean index score (SD)	0.674 (0.246)	0.655 (0.234)	0.010 (−0.049 to 0.069); p=0.75
Mean VAS score (SD)	71.79 (16.29)	66.72 (20.14)	5.60 (0.51 to 10.85); p=0.033
**EQ-5D-5L12months**			
n	74	76	
Mean index score (SD)	0.690 (0.233)	0.638 (0.277)	0.024 (−0.039 to 0.084); p=0.45
Mean VAS score (SD)	71.22 (18.81)	62.67 (22.93)	7.96 (1.45 to 14.05); p=0.013
**QALYs**			
n	73	76	
Unadjusted mean (SD)	0.682 (0.205)	0.646 (0.203)	0.036 (−0.032 to 0.102); p=0.30
Adjusted mean (SD)			0.013 (−0.033 to 0.052); p=0.56

Utility scores were replaced with zero for deaths.

* Bootstrapped differences are adjusted for baseline EQ-5D-5L, modified European Heart Rhythm Association class, left ventricular ejection fraction, age and gender; values >0 favour digoxin in comparison to beta-blockers.

EQ-5D-5L, EuroQol 5-Domain 5-Level instrument; QALY, quality-adjusted life year; VAS, visual analogue scale.

### Economic evaluation

The complete case data (costs and QALYs) from 149 patients showed that digoxin was associated with a significantly lower total cost: adjusted difference −£530.41 (95% CI −£848.06 to −£249.38, p=0.001) with no significant difference in QALYs (adjusted difference 0.013, 95% CI −0.033 to 0.052, p=0.056). Therefore, beta-blockers were dominated by digoxin. The result did not change when all randomised patients with imputed missing values were included (table 4).

**Table 4:** Results of the cost-utility analysis from the healthcare perspective

Cost-utility	Mean per-patient total costs, 12 months following randomisation (£) (SD)	Mean bootstrapped per-patient cost (£) difference* (95% CI) and P value for digoxin versus beta-blockers	Mean QALYs (SD)	Mean bootstrapped QALY difference† (95% CI) and P value for digoxin versus beta-blockers	Incremental cost-effectiveness ratio (£/QALY)
**Base case—completed resource use and EQ-5D data**					
Beta-blockers (n=76)	535.39 (1261.21)	−530.41 (−848.06 to −249.38); p=0.001	0.646 (0.203)	0.013 (−0.033 to 0.052); p=0.56	Digoxin dominant
Digoxin (n=73)	46.19 (87.19)		0.682 (0.205)		
**Sensitivity analysis—imputed data** ‡					
Betablockers (n=80)	526.90 (1231.33)	−418.32 (−744.26 to −150.68); p=0.57	0.638 (0.203)	0.012 (−0.028 to 0.053); p=0.006	Digoxin dominant
Digoxin (n=80)	137.59 (759.15)		0.673 (0.211)		

* Adjusted for baseline modified European Heart Rhythm Association class, left ventricular ejection fraction, age and gender.

† Adjusted for baseline EQ-5D-5L, modified European Heart Rhythm Association class, left ventricular ejection fraction, age and gender.

‡ A cost outlier patient with missing EQ-5D-5L data was included here in the digoxin arm.

EQ-5D-5L, EuroQol 5-Domain 5-Level; QALY, quality-adjusted life year.

**Figure 2:**
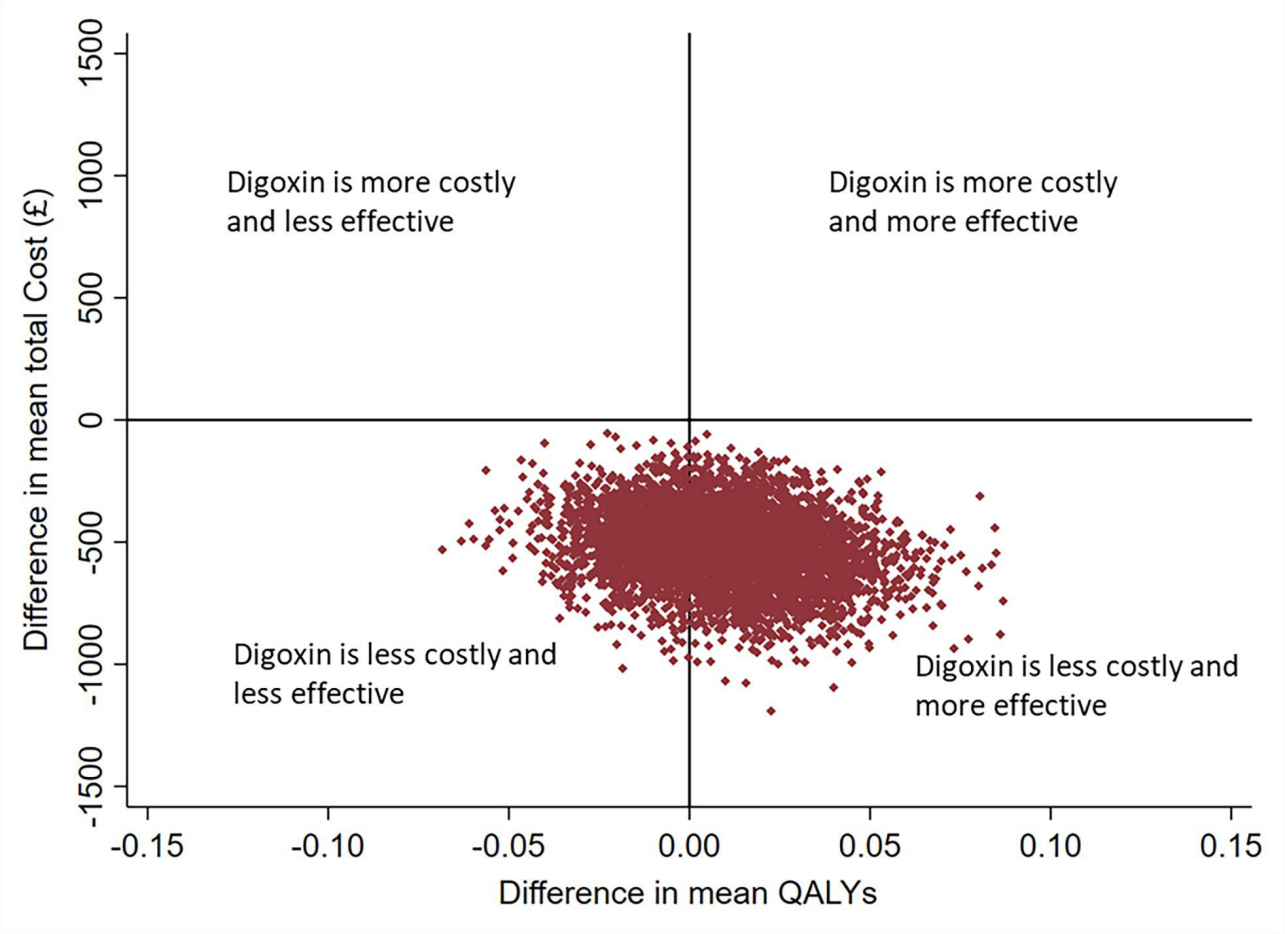
Cost-effectiveness plane for digoxin versus beta-blockers. Adjusted differences in costs and quality-adjusted life years (QALYs) from the UK National Health Service perspective over a 12-month period.

**Figure 3:**
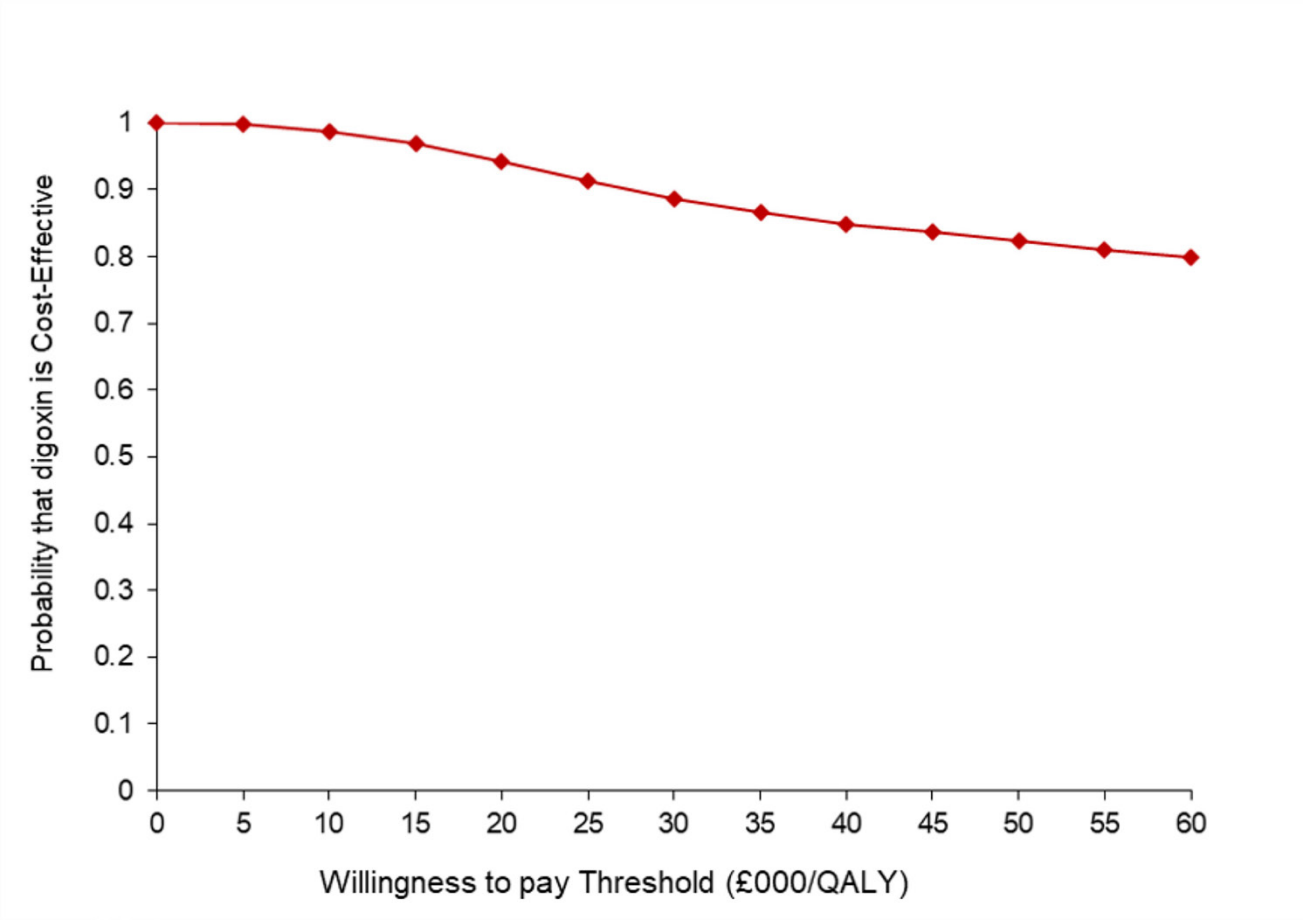
Cost-effectiveness acceptability curve. Indicates the probability of digoxin being cost-effective compared to beta-blockers across different willingness-to-pay thresholds per additional quality-adjusted life year (QALY) at 12 months from the UK National Health Service perspective.

Results of the 5000 generated bootstrap estimates, presented on the cost-effectiveness plane (figure 2), also supported the findings of the base case analysis. Most of the incremental costs and QALY pairs are in the south-east quadrant and with the remaining points in the south-west quadrant. This can be interpreted as lower healthcare costs in the digoxin arm with uncertainty in the direction of the QALY difference. The cost-effectiveness acceptability curve in figure 3 shows that digoxin was 94% and 89% cost-effective at willingness-to-pay thresholds per QALY gained of £20 000 and £30 000, respectively. Post hoc extrapolation to current UK NHS prevalence and cost for AF suggests a saving of £102 million/annum (95% CI £48 million to £164 million, p=0.001), equivalent to a 5.9% saving on the annual budget spent on AF in the UK (online supplemental table 4).

## Discussion

This economic evaluation indicates that digoxin is a cost-saving intervention over 12 months for heart rate control in patients with permanent AF, compared with standard beta-blocker therapy. Digoxin dominated beta-blockers in this common group of patients with symptoms of heart failure, and there is a high probability of cost-effectiveness at the NICE threshold of £20 000 per QALY gained. The significantly lower cost of digoxin was related to fewer SAEs, less complicated care, lower rates of hospital stay and fewer GP and nurse contacts, compared with beta-blockers. This analysis was based on a single RCT; however, extrapolation to routine healthcare suggests substantial healthcare savings for an increasingly prevalent and costly condition.

Over 37 million people had a diagnosis of AF globally in 2017, an increase of 33% since 1997.^22^ The prevalence of AF is expected to escalate further due to new technologies than can detect AF.^23^ In the USA, the total healthcare cost for each patient with AF was estimated at $63 031 per year, compared with $35 135 per year for those without AF.^24^ In the UK, the total cost of AF is predicted to increase from £1741 million in 2020 (1.1% of NHS expenditure) to £5668 million by 2040 (2.0% of NHS expenditure).^3^ This will be further compounded by health and social care costs for sequelae such as vascular dementia, which is associated with AF even in patients without conventional stroke risk factors.^1^ Economic analysis is a critical component of ensuring that health services can meet fiscal restrictions while ensuring optimal patient care. Previous economic analyses in AF have largely focused on anticoagulant therapy to prevent stroke, screening approaches for AF and interventional approaches such as catheter ablation.^25^,^27^ In a systematic review of 50 studies,^28^ only a single study evaluated the cost-effectiveness of different rate and rhythm control drugs^29^; cost-effectiveness varied considerably, although this may be due to observational prescription biases in the Korean health insurance database used.

To our knowledge, this is the first trial-based economic evaluation conducted to compare different therapeutic approaches for heart rate control in patients with AF. Data on healthcare resource use at different levels were collected prospectively within an RCT, with collation of adverse events to map the complexity of care in this multimorbid patient group. The actual resultant costs from resource use were therefore measured, in contrast to estimation that would be typical for a model-based economic evaluation. Embedding within a trial was essential as digoxin is typically used as a second-line medication for older, more comorbid patients or those who have already failed to respond to beta-blockers. These factors are all linked to worse outcomes and higher healthcare need.^30^

The key limitation of this analysis is the sample size of the RATE-AF trial, leading to uncertainty in results. However, no other direct comparison RCTs have been performed for digoxin therapy. A microcosting analysis conducted alongside a trial to collect detailed resource use data on high-cost events can lead to more accurate cost estimates. However, this approach may be impractical in clinical trials due to the demands on time, resources and the data collection effort required. Although 12 months is a short period to capture long-term outcomes associated with AF, the participants in the trial had AF for an average of 3.8 years prior to enrolment (95% CI 2.8 to 4.7), and so the study period was a good reflection of this patient group. The RATE-AF trial was not designed or powered to detect differences in cardiovascular events, although comparing total adverse events with digoxin versus beta blockers was a prespecified secondary outcome. The level of data completion was high, and findings did not change when a multiple imputation approach was employed to replace missing values. In order to support policy-making on the local, national and global scales, further research is clearly warranted. This should include comparative rate control trials with prespecified economic analyses to identify the most cost-effective interventions for different patient scenarios, and to ensure generalisability of results. A decision analytical model would be beneficial to explore the cost-effectiveness of alternative interventions over a longer time horizon.

## Conclusion

Low-dose digoxin is a cost-effective intervention for heart rate control in patients with permanent AF compared with the usual clinical approach using beta-blockers. Use of digoxin could lead to substantial cost savings, with further and longer term research needed in view of the limited sample size and uncertainty around improvement in QoL.

## Supplementary material

10.1136/heartjnl-2024-324761 online supplemental file 1

10.1136/heartjnl-2024-324761 Uncited online supplemental file 2

## Data Availability

Data may be obtained from a third party and are not publicly available.

## References

[1] Mobley AR, Subramanian A, Champsi A (2024). Thromboembolic events and vascular dementia in patients with atrial fibrillation and low apparent stroke risk. N Med.

[2] Lane DA, Skjøth F, Lip GYH (2017). Temporal Trends in Incidence, Prevalence, and Mortality of Atrial Fibrillation in Primary Care. J Am Heart Assoc.

[3] Burdett P, Lip GYH (2022). Atrial fibrillation in the UK: predicting costs of an emerging epidemic recognizing and forecasting the cost drivers of atrial fibrillation-related costs. Eur Heart J Qual Care Clin Outcomes.

[4] Kotecha D, Calvert M, Deeks JJ (2017). A review of rate control in atrial fibrillation, and the rationale and protocol for the RATE-AF trial. BMJ Open.

[5] Hagens VE, Vermeulen KM, TenVergert EM (2004). Rate control is more cost-effective than rhythm control for patients with persistent atrial fibrillation--results from the RAte Control versus Electrical cardioversion (RACE) study. Eur Heart J.

[6] Ţica O, Khamboo W, Kotecha D (2022). Breaking the Cycle of Heart Failure With Preserved Ejection Fraction and Atrial Fibrillation. Card Fail Rev.

[7] Bunting KV, Stanbury M, Tica O (2021). Transforming clinical research by involving and empowering patients- the RATE-AF randomized trial. Eur Heart J.

[8] Kotecha D, Bunting KV, Gill SK (2020). Effect of Digoxin vs Bisoprolol for Heart Rate Control in Atrial Fibrillation on Patient-Reported Quality of Life: The RATE-AF Randomized Clinical Trial. JAMA.

[9] National Institute for Health and Care Excellence (NICE) Guide to the methods of technology appraisal 2013. www.nice.org.uk/article/pmg9/chapter/foreword.

[10] Drummond MF, Sculpher MJ, Claxton K (2015). Methods for the economic evaluation of health care programmes.

[11] National schedule of reference costs 2019/2020 for NHS Trusts. https://www.england.nhs.uk/publication/2019-20-national-cost-collection-data-publication/.

[12] Manca A, Hawkins N, Sculpher MJ (2005). Estimating mean QALYs in trial-based cost-effectiveness analysis: the importance of controlling for baseline utility. Health Econ.

[13] Barber JA, Thompson SG (2000). Analysis of cost data in randomized trials: an application of the non-parametric bootstrap. Stat Med.

[14] Fenwick E, Claxton K, Sculpher M (2001). Representing uncertainty: the role of cost-effectiveness acceptability curves. Health Econ.

[15] Burton A, Billingham LJ, Bryan S (2007). Cost-effectiveness in clinical trials: using multiple imputation to deal with incomplete cost data. Clin Trials.

[16] White IR, Royston P, Wood AM (2011). Multiple imputation using chained equations: Issues and guidance for practice. Stat Med.

[17] Chiang C-E, Naditch-Brûlé L, Murin J (2012). Distribution and risk profile of paroxysmal, persistent, and permanent atrial fibrillation in routine clinical practice: insight from the real-life global survey evaluating patients with atrial fibrillation international registry. Circ Arrhythm Electrophysiol.

[18] Rubin DB, Schenker N (1991). Multiple imputation in health-care databases: an overview and some applications. Stat Med.

[19] (2023). British Heart Foundation 2023 statistics; based on 2021/22 primary care register data (all UK). Heart and circulatory disease statistics 2023.

[20] Santhanakrishnan R, Wang N, Larson MG (2016). Atrial Fibrillation Begets Heart Failure and Vice Versa: Temporal Associations and Differences in Preserved Versus Reduced Ejection Fraction. Circulation.

[21] Phillips K, Subramanian A, Thomas GN (2022). Trends in the pharmacological management of atrial fibrillation in UK general practice 2008-2018. Heart.

[22] Lippi G, Sanchis-Gomar F, Cervellin G (2021). Global epidemiology of atrial fibrillation: An increasing epidemic and public health challenge. Int J Stroke.

[23] Gill S, Bunting KV, Sartini C (2022). Smartphone detection of atrial fibrillation using photoplethysmography: a systematic review and meta-analysis. Heart.

[24] Deshmukh A, Iglesias M, Khanna R (2022). Healthcare utilization and costs associated with a diagnosis of incident atrial fibrillation. H R O.

[25] Noviyani R, Youngkong S, Nathisuwan S (2022). Economic evaluation of direct oral anticoagulants (DOACs) versus vitamin K antagonists (VKAs) for stroke prevention in patients with atrial fibrillation: a systematic review and meta-analysis. BMJ Evid Based Med.

[26] van Hulst M, Tieleman RG, Zwart LAR (2023). Health economic evaluation of nation-wide screening programmes for atrial fibrillation in the Netherlands. Eur Heart J Qual Care Clin Outcomes.

[27] Chew DS, Loring Z, Anand J (2020). Economic Evaluation of Catheter Ablation of Atrial Fibrillation in Patients with Heart Failure With Reduced Ejection Fraction. Circ Cardiovasc Qual Outcomes.

[28] Okafor C, Byrnes J, Stewart S (2023). Cost Effectiveness of Strategies to Manage Atrial Fibrillation in Middle- and High-Income Countries: A Systematic Review. Pharmacoeconomics.

[29] Kim M, Kim W, Kim C (2019). Cost-Effectiveness of Rate- and Rhythm-Control Drugs for Treating Atrial Fibrillation in Korea. Yonsei Med J.

[30] Champsi A, Mitchell C, Tica O Digoxin in patients with heart failure and/or atrial fibrillation: a systematic review and meta-analysis of 5.9 million patient years of follow-up. SSRN.

