# Supplementary material for: Cost-effectiveness of digoxin versus beta blockers in permanent atrial fibrillation: the Rate Control Therapy Evaluation in Permanent Atrial Fibrillation (RATE-AF) randomised trial
Source: Heart. 2025 Jan 16;111(8):e324761. doi: 10.1136/heartjnl-2024-324761 (PMC12015011; doi:10.1136/heartjnl-2024-324761)
Supplement: online supplemental file 1 [file heartjnl-111-8-s001.docx]

**Cost-effectiveness of digoxin versus beta-blockers in permanent atrial fibrillation: the RATE-AF randomised trial**

Zainab Abdali MSc, Karina V Bunting PhD, Samir Mehta MSc, A John Camm MD, Kazem Rahimi DM, Mary Stanbury, Sandra Haynes MBE, Dipak Kotecha PhD, Sue Jowett PhD, on behalf of the Rate control Therapy Evaluation in permanent Atrial Fibrillation (RATE-AF) trial team.

**SUPPLEMENT CONTENTS:**

[Supplementary Table 1: Eligibility criteria for the RATE-AF trial 2](#_Toc169608890)

[Supplementary Table 2: Resource use unit costs (GBP 2019/20 prices) 3](#_Toc169608891)

[Supplementary Table 3: Clinical events by randomized group 5](#_Toc169608892)

[Supplementary Table 4: Extrapolation of savings to the UK National Health Service 6](#_Toc169608893)

[References for supplement 7](#_Toc169608894)

**Supplementary Table 1: Eligibility criteria for the RATE-AF trial**

| **Inclusion criteria** |
| --- |
| Adult patients aged 60 years or older. |
| Permanent AF, characterised (at time of randomisation) as a physician decision for rate-control with no plans for cardioversion, anti-arrhythmic medication, or ablation therapy. |
| Symptoms of breathlessness (New York Heart Association Class II or more). |
| Able to provide written informed consent. |
| **Exclusion criteria** |
| Established clinical indication for beta-blocker therapy, e.g. myocardial infarction in the last 6 months. |
| Known contraindications for therapy with beta blocker or digoxin e.g. a history of severe bronchospasm that would preclude use of beta-blockers, or known intolerance to these medications. |
| Baseline heart rate less than 60 beats per minute. |
| History of second- or third-degree block. |
| Supraventricular arrhythmias associated with accessory conducting pathways (e.g. Wolff-Parkinson-White syndrome) or a history of ventricular tachycardia or fibrillation. |
| Planned pacemaker implantation (including cardiac resynchronisation therapy), pacemaker-dependent rhythm or history of atrioventricular node ablation. |
| Decompensated heart failure (evidenced by need for intravenous inotropes, vasodilators or diuretics) within 14 days prior to randomisation. |
| A current diagnosis of obstructive hypertrophic cardiomyopathy, myocarditis or constrictive pericarditis. |
| Received or on waiting list for heart transplantation. |
| Receiving renal replacement therapy. |
| Major surgery, including thoracic or cardiac surgery, within 3 months of randomisation. |
| Severe, concomitant non-cardiovascular disease (including malignancy) that is expected to reduce life expectancy. |

**Supplementary Table 2: Resource use unit costs (GBP 2019/20 prices)**

| Resource use item | Cost | Cost per unit | Description | Source |
| --- | --- | --- | --- | --- |
| **Primary healthcare services** | | | | |
| GP at practice | £39.23/visit | £39.23/visit | 9.22 minutes contact | ^1^ |
| GP home visit | £4.30/minute | £100.62/visit | 11.4 minutes consultation + 12 minutes travel | ^1^ |
| Nurse at practice | £42/hour | £10.85/visit | 15.5 minutes contact | ^1^ |
| Nurse home visits | £42/hour | £17.50/visit | 25 minutes including travel time | ^1^ |
| **Secondary healthcare services (inpatient care)** | | | | |
| Heart failure |  | £4,057/episode | Heart Failure or Shock, with CC Score 14+ (non-elective long stay) for HRG (EB03A) | ^2^ |
|  |  | £2,786/episode | Average cost of Heart Failure or Shock, (Non-elective long stay) for HRGs (EB03A-E) | ^2^ |
|  |  | £523/episode | Average cost of Heart Failure or Shock (day case) for HRGs (EB03A-E) | ^2^ |
| Arrythmia |  | £3,406/episode | Arrhythmia or Conduction Disorders, with CC Score 13+ (nonelective short stay) for HRG (EB07A) | ^2^ |
|  |  | £648/episode | Average cost of Arrhythmia or Conduction Disorders (nonelective short stay) for HRGs (EB07A-E) | ^2^ |
| Chest pain |  | £405/episode | Average cost of Unspecified Chest Pain (Nonelective - short stay) for HRGs (EB12A-C) | ^2^ |
| Stroke |  | £4,717/episode | Average cost of stroke (Nonelective long stay) for HRGs (AA35A-F) | ^2^ |
| Angioplasty |  | £2,726/episode | Average cost of Standard Percutaneous Transluminal Coronary Angioplasty (Nonelective-short stay) for HRGs (EY41 A-D) | ^2^ |
| Endocarditis |  | £1,613/episode | Average cost of endocarditis (Nonelective-short stay) for HRGs (EB02A-C) | ^2^ |
| Myocardial infarction |  | £1,249/episode | Average of actual or suspended MI (Day case) for HRGs (EB10A-E) | ^2^ |
| Syncope |  | £3,286/episode | Syncope or Collapse, with CC Score 13+ (Nonelective-long stay) for HRG EB08A | ^2^ |
| Dual chamber pacemaker |  | £2,839/procedure | Average cost of Pacemaker implementation (dual chamber) (Nonelective - short stay for HRGs (EY06A-E) | ^2^ |
|  |  | £1,927/procedure | Average of Pacemaker implementation (dual chamber) (day case) for HRGs (EY06A-E) | ^2^ |
| Single chamber pacemaker |  | £1,726/procedure | Average of implantation of Single-Chamber Pacemaker (day case) for HRGs (EY08A-E) | ^2^ |
| **Medication** | | | | |
| Trial medication: | | | | |
| Generic digoxin 125 μg | £1.70 /pack | £0.06/tablet | Pack of 28 tabs | ^3^ |
| Generic bisoprolol 3.75mg | £1.23 /pack | £0.04/tablet | Pack of 28 tabs | ^3^ |
| Additional medication: | | | | |
| Diltiazem 120 – 300 mg | £5.49 - £6.98 /pack | £0.20 - £0.25/tablet | Pack of 28 caps | ^3^ |

CC, Clinical Classification; GBP, Great Britain pounds sterling; GP, General Practitioner; HRG, Healthcare Resource Group.

# Supplementary Table 3: Clinical events by randomized group

| Outcome | Digoxin (n=80) | Beta-blocker (n=80) |
| --- | --- | --- |
| ***Deaths*** | | |
| Number (%) | 4 (5.0%) ^a^ | 7 (8.8%) ^b^ |
| ***Adjudicated cardiovascular events ^c^*** | | |
| Total number | 3 (in 2 patients) ^d^ | 15 (in 12 patients) ^e^ |
| ***Unplanned hospitalizations*** | | |
| Total number | 12 (in 11 patients) | 28 (in 19 patients) |
| Number with two or more hospital admissions | 1 | 9 |
| ***Serious adverse events ^f^*** | | |
| Total number | 16 (in 13 patients) | 37 (in 21 patients) |
| ***Treatment-related adverse events ^g^*** | | |
| Total number | 29 | 142 |
| Number (%) with at least one event | 20 (24.7%) | 51 (63.8%) |
| ***Primary care visits in addition to study visits^h^*** | | |
| Total number of visits | 192 (in 64 patients) | 228 (in 68 patients) |
| Number of visits due to atrial fibrillation | 6 (in 4 patients) | 30 (in 21 patients) |
| Number of visits due to other cardiovascular cause | 16 (in 9 patients) | 34 (in 23 patients) |
| Number of visits due to non- cardiovascular or other cause | 170 (in 61 patients) | 164 (in 58 patients) |

^a^ Causes of death were ischemic heart disease, bladder cancer, aspiration pneumonia in the context of colon cancer, and liver cirrhosis in the context of alcoholic liver disease. ^b^ Causes of death were congestive cardiac failure, decompensated heart failure in the context of severe valve disease, non-Hodgkin’s lymphoma, cardio-renal syndrome, myocardial infarction, pancreatic cancer, and perforated bowel secondary to diverticular disease. ^c^ For any potential cardiovascular event, an independent clinician reviewed medical records, blood results and imaging, and completed a pre-specified structured case report form that was sent directly to the trials unit. ^d^ Primary causes were myocardial infarction, peripheral oedema after diuretics were inadvertently paused, and palpitations with no change to management. ^e^ Primary causes were pacemaker implantation x 2 (bradycardia and/or pauses), decompensated heart failure x 3, myocardial infarction x 2, troponin-negative chest pain x 2, acute stroke x 2, collapse and bradycardia, heart failure and bradycardia, rapid AF and dyspnoea, and endocarditis. ^f^ Serious adverse events are any adverse event, adverse reaction or unexpected adverse reaction, respectively, that results in death, is life-threatening, requires hospitalization or prolongation of existing hospitalization, results in persistent or significant disability or incapacity, or consists of a congenital anomaly or birth defect; all such events underwent appraisal by a Principal Investigator within one working day, followed by confirmatory processes by the Chief Investigator. ^g^ At each study visit, patients were asked to report any adverse events since the last visit from a list taken from the Summary of Product Characteristics for each drug. ^h^ On average, there were 3.2 primary care contacts per patient in addition to trial visits; in a national survey in Scotland, the average number of contacts per patient (with newly diagnosed AF) was between 4.2 and 7.8.^4^

**Supplementary Table 4: Extrapolation of savings to the UK National Health Service**

| Row | Component | n, % or £GBP | Source |
| --- | --- | --- | --- |
| 1 | Number in UK with AF | 1,519,109 | ^5^ |
| 2 | Proportion of AF with permanent AF | 49.6% | ^6^ |
| 3 | Number in UK with permanent AF | 753,478 | *Calculated* |
| 4 | HF diagnosis in patients with AF | 36.6% | ^7^ |
| 5 | Number in UK with permanent AF + HF | 275,773 | *Calculated* |
| 6 | Use of rate control in permanent AF | 84.2% | ^6^ |
| 7 | Proportion already using digoxin for rate control | 16.9% | ^8^ |
| 8 | Number in UK with permanent AF + HF + rate control | 192,959 | *Calculated* |
| 9 | Digoxin saving vs beta-blocker, per-patient, per-year | £530 | RATE-AF health economic analysis |
| **10** | **Digoxin saving vs beta-blocker, UK per-year** | **£102,347,330** | ***Calculated*** |
| 11 | 2020 NHS cost for AF | £1,741,000,000 | ^9^ |
| **12** | **Potential saving as proportion of annual NHS AF budget** | **5.9%** | ***Calculated*** |

AF, atrial fibrillation; GBP, Great Britain pounds sterling; HF, heart failure; n, number of participants; NHS, National Health Service.

# References for supplement

1. Curtis L, Burns A. Unit costs of health and social care 2020. Canterbury. Personal Social Services Research Unit University of Kent. 2020

2. National schedule of reference costs 2019/2020 for NHS Trusts. Available at:<https://www.england.nhs.uk/publication/2019-20-national-cost-collection-data-publication/> (accessed 10th Novmber 2021).

3. British National Formulary (BNF). Available at: <https://bnf.nice.org.uk/drugs> (accessed 11th November 2021).

4. Murphy NF, Simpson CR, Jhund PS*, et al.* A national survey of the prevalence, incidence, primary care burden and treatment of atrial fibrillation in Scotland. *Heart*. 2007;93:606-612

5. British Heart Foundation 2023 statistics; based on 2021/22 primary care register data (all UK). Heart and circulatory disease statistics 2023 - BHF UK statistics Aug 2023

6. Chiang C-E, Naditch-Brûlé L, Murin J*, et al.* Distribution and risk profile of paroxysmal, persistent, and permanent atrial fibrillation in routine clinical practice: insight from the real-life global survey evaluating patients with atrial fibrillation international registry. *Circulation: Arrhythmia and Electrophysiology*. 2012;5:632-639

7. Santhanakrishnan R, Wang N, Larson MG*, et al.* Atrial fibrillation begets heart failure and vice versa: temporal associations and differences in preserved versus reduced ejection fraction. *Circulation*. 2016;133:484-492

8. Phillips K, Subramanian A, Thomas GN*, et al.* Trends in the pharmacological management of atrial fibrillation in UK general practice 2008–2018. *Heart*. 2022;108:517-522

9. Burdett P, Lip GY. Atrial fibrillation in the UK: predicting costs of an emerging epidemic recognizing and forecasting the cost drivers of atrial fibrillation-related costs. *European Heart Journal-Quality of Care and Clinical Outcomes*. 2022;8:187-194
