# Supplementary material for: Cost-effectiveness of digoxin versus beta blockers in permanent atrial fibrillation: the Rate Control Therapy Evaluation in Permanent Atrial Fibrillation (RATE-AF) randomised trial
Source: Heart. 2025 Jan 16;111(8):e324761. doi: 10.1136/heartjnl-2024-324761 (PMC12015011; doi:10.1136/heartjnl-2024-324761)
Supplement: Uncited online supplemental file 2 [file heartjnl-111-8-s002.pdf]

Cost-effectiveness of low-dose digoxin versus beta-blockers for heart rate control in permanent atrial fibrillation

RATE AF

Methods and participants

Randomised, open-label, NHS embedded trial

160 patients (permanent AF and syndrome of heart failure)

Women **46%**

Mean age **76 ± 8** years

A **trial-based** health economic analysis from the perspective of the **NHS**

Patient-level data on resource use and health outcome collected at baseline, 6 months and 12 months

Economic evaluation results

Annual NHS costs per patient

Digoxin

£46.19

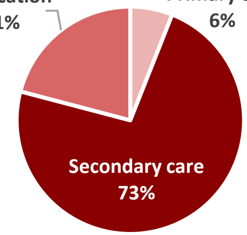

Beta-blocker

£535.39

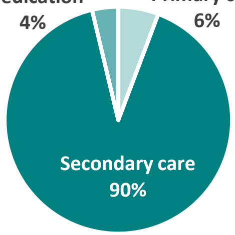

£530.41* per patient likely to be saved if digoxin is used

NHS could potentially save **£102 million/year** by implementing digoxin to control heart rate in patients with permanent atrial fibrillation

*Difference adjusted for baseline characteristics

Primary outcome **0.013** (95% CI, -0.033 to 0.052) additional Quality Adjusted Life Year (QALY) with Digoxin

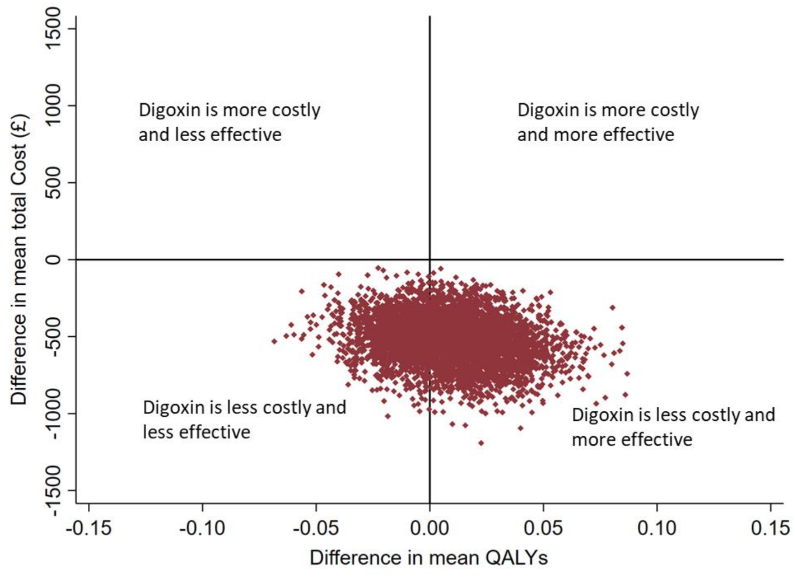

Cost-effectiveness plane for Digoxin and beta-blocker

◆ Each point represents 1 bootstrapped iteration of difference in costs and QALYs

Low-dose digoxin is a less costly intervention compared to beta-blockers for heart rate control in permanent atrial fibrillation
